# Rim plate in the treatment of hyperextension tibial plateau fracture: surgical technique and a series of cases

**DOI:** 10.1186/s12891-023-06786-z

**Published:** 2023-08-17

**Authors:** Zhijian Sun, Ting Li, Yabo Liu, Yujiang Mao, Weihua Li, Qi Guo, Shaoliang Li, Changrun Li

**Affiliations:** Department of Orthopedic Trauma, Beijing Jishuitan Hospital, Beijing, 100035 PR China

**Keywords:** Tibial plateau fracture, Hyperextension, Rim plate

## Abstract

**Background:**

The existence of a “bare area” at the anterior plateau has been observed in cases where anteromedial and/or anterolateral proximal tibial locking plates are used for fixation in the treatment of hyperextension tibial plateau fractures (HTPF). The objective of this study is to introduce the rim plate fixation technique and evaluate its clinical efficacy.

**Methods:**

A retrospective analysis was conducted on HTPF patients who underwent treatment with a combination of rim plate and proximal tibial locking plate at our hospital between April 2015 and December 2019. All patients were followed up for a minimum of one year. Open reduction and internal fixation were performed using anteromedial/posteromedial and/or anterolateral approaches for all cases. The surgical strategies employed for rim plate fixation were introduced, and both radiographic and clinical outcomes were assessed.

**Results:**

Thirteen patients were enrolled in the study, with an average follow-up time of 4.3 years. Satisfactory reduction was achieved and radiographically maintained in all cases. Additionally, all patients exhibited satisfactory clinical functions, as evidenced by a mean hospital for special surgery (HSS) knee score of 96.2 ± 2.0 (range: 90–98). Furthermore, no wound complications or implant breakage were observed in this series.

**Conclusion:**

The combination of the rim plate and proximal tibial plate proved to be an effective fixation configuration, resulting in satisfactory clinical outcomes.

## Background

Computed tomography (CT) and magnetic resonance imaging (MRI) are frequently employed for preoperative assessment, and numerous authors have utilised them to elucidate the mechanisms of tibial plateau fractures [1, 2]. The sagittal plane elaborates on the direction of forces, encompassing hyperextension, extension, and flexion types; the coronal plane involves varus and valgus forces, while the horizontal plane includes rotation and transition. Hyperextension tibial plateau fractures (HTPF) exhibit an anterior depression or collapse of the medial and/or lateral plateau, with more severe involvement when both condyles are fractured [3]. Firoozabadi et al. [4], in their study, demonstrated a high incidence of popliteal artery disruption, peroneal nerve injury, and compartment syndrome in hyperextension tibial plateau bicondylar fractures compared to other bicondylar plateau fractures, which was further verified by later studies [5, 6]. Furthermore, the clinical outcome of HTPF was found to be inferior, with worse functional outcomes [7, 8].

**Fig. 1:**
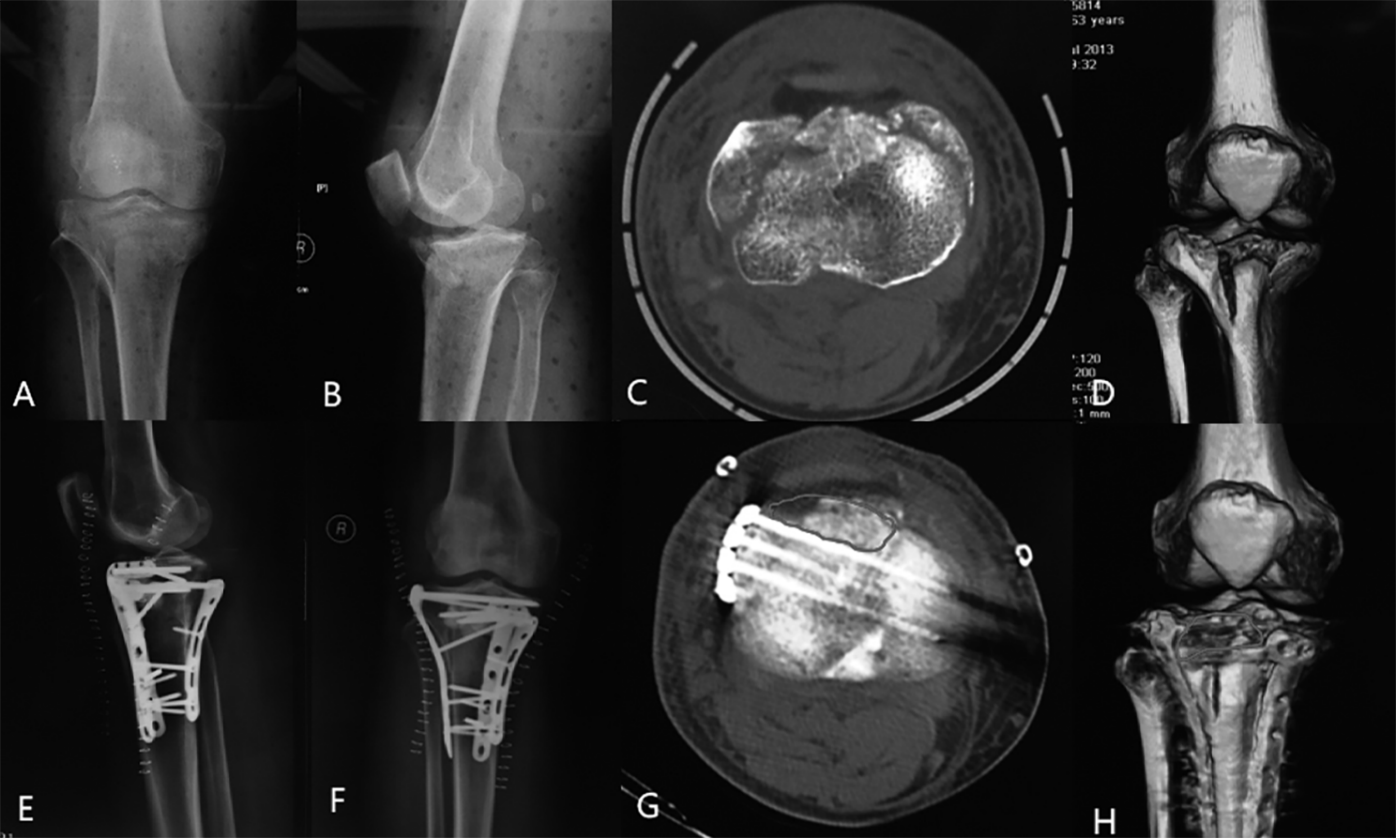
A 63-year-old male patient presented with bicondylar HTPF **(A-D)**. Subsequently, open reduction and fixation using anteromedial and anterolateral locking plates were performed **(E, F).** Postoperative CT scans **(G, H)** revealed the “buttress bare area” behind the patella tendon (indicated by grey circles)

The fixation of reduced HTPF commonly involves the use of anteromedial and/or anterolateral proximal tibial locking plates. However, there exists a challenging “bare area” at the anterior plateau between these two plates (Fig. 1). This region poses difficulties for fixation due to the shelter of the patella tendon and limited space for plate placement. To address this issue, our trauma center developed the 2.7 mm pre-contoured rim plate to buttress the anterior plateau. The rim plate is primarily employed for fixing the posterolateral plateau, as it offers better fixation strength against anti-posterior displacement than the standard lateral locking plate [9, 10]. Notably, no prior study has reported on the usage of rim plates for treating HTPF. In this study, we present the surgical strategies involving rim plates for HTPF treatment and the preliminary results of a case series.

## Methods

### Patients and preoperative evaluation

Patients diagnosed with HTPF and treated with rim plates between April 2015 and December 2019 were subjected to a retrospective analysis. All patients were followed up for a minimum of one year. HTPF was characterized by the medial and/or lateral depression of the anterior plateau along with a reduced posterior slope angle (PSA). Patients with pathological and chronic fractures were excluded from the study, as were those with multiple injuries or accompanying fractures. The ethical committee of Beijing Jishuitan Hospital approved this study (Approval No. 202111-13).

Upon emergency admission, all patients underwent temporary immobilization using casts, braces, or external fixators. Subsequently, preoperative radiographs and CT scans were obtained for all patients to assess the morphological characteristics of the fracture. The definitive surgical treatment, involving open reduction and internal fixation, was performed when the soft tissue condition allowed, indicated by the fading of blisters and improvement in swelling, as evaluated by the surgeon.

### Surgical technique

Patients with fractures solely involving the medial or lateral plateau underwent a single anteromedial or anterolateral approach. For those with bicondylar fractures, the most common approach involved anteromedial/posteromedial and anterolateral approaches. Bicondylar HTPF patients typically presented with posterior tension fractures, wherein the posteromedial incision was initially made to expose and reduce the posterior fracture. Subsequently, a 1/3 tubular plate or anatomical plate with a short proximal screw was utilized to facilitate minimal transition during the elevation of the anterior decompression, creating a hinge point. Next, reduction of the anterior depressed articular surface to restore the sagittal and coronal alignments was performed mainly following the approach described by Firoozabadi et al. [4]. This involved inserting an osteotome or spreader into the impaction area of the anterior metaphysis and elevating it until the PSA was restored. The use of multiple Kirschner wires or plates for reduction was also found to be an effective method. The same reduction procedure was applied for HTPF patients with fractures solely involving the medial or lateral plateau. Once satisfactory reduction was achieved and verified by intraoperative fluoroscopy, provisional stabilization was performed using Kirschner wires. Structural artificial bone augments (MIIG™ X3 or PRO-DENSE™ Injectable, Wright Medical Technology, Inc., Memphis, USA) were employed if the anterior defect exceeded 1 cm.

**Fig. 2:**
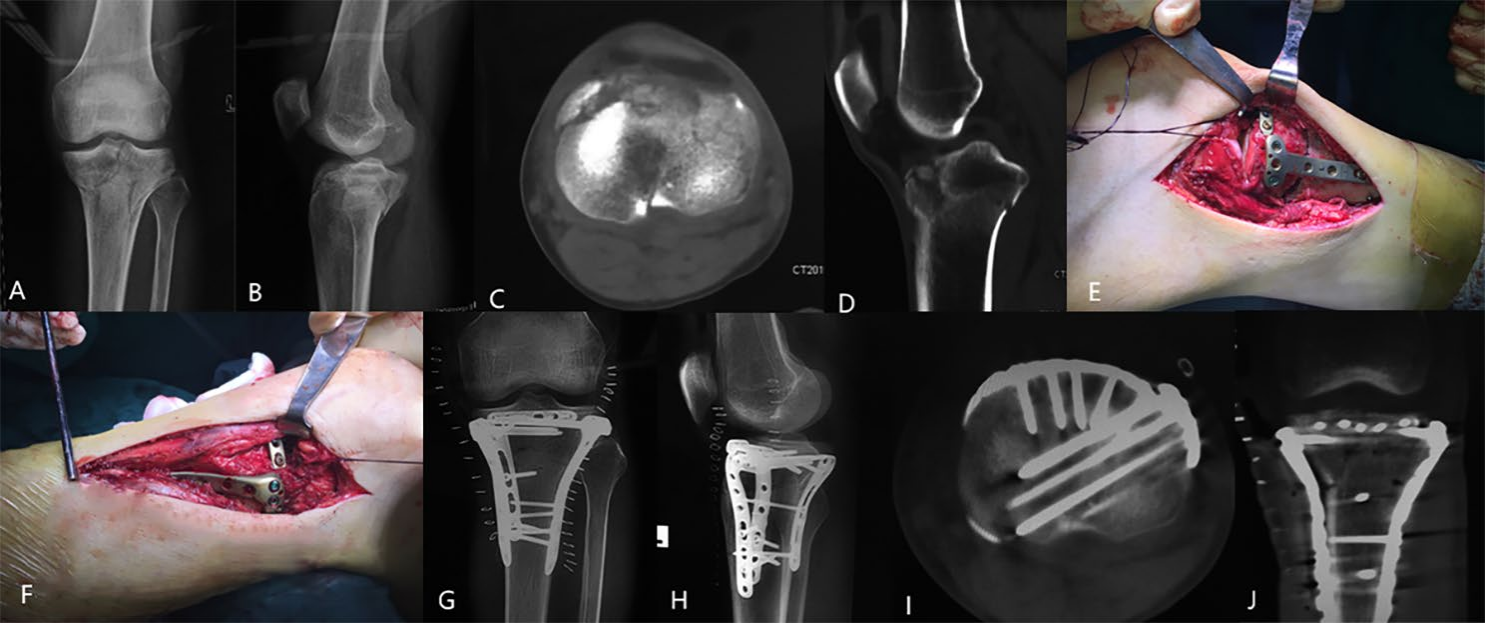
A 39-year-old male patient presented with bicondylar HTPF **(A-D)**. To address this, a combination of the rim plate and proximal tibial locking plates was employed **(E-H)**. The rim plate was strategically inserted behind the patella tendon, with screws placed through lifting both sides of the patella **(E, F)**. Subsequent postoperative CT scans revealed adequate support provided to the anterior plateau **(I, J)**

**Fig. 3:**
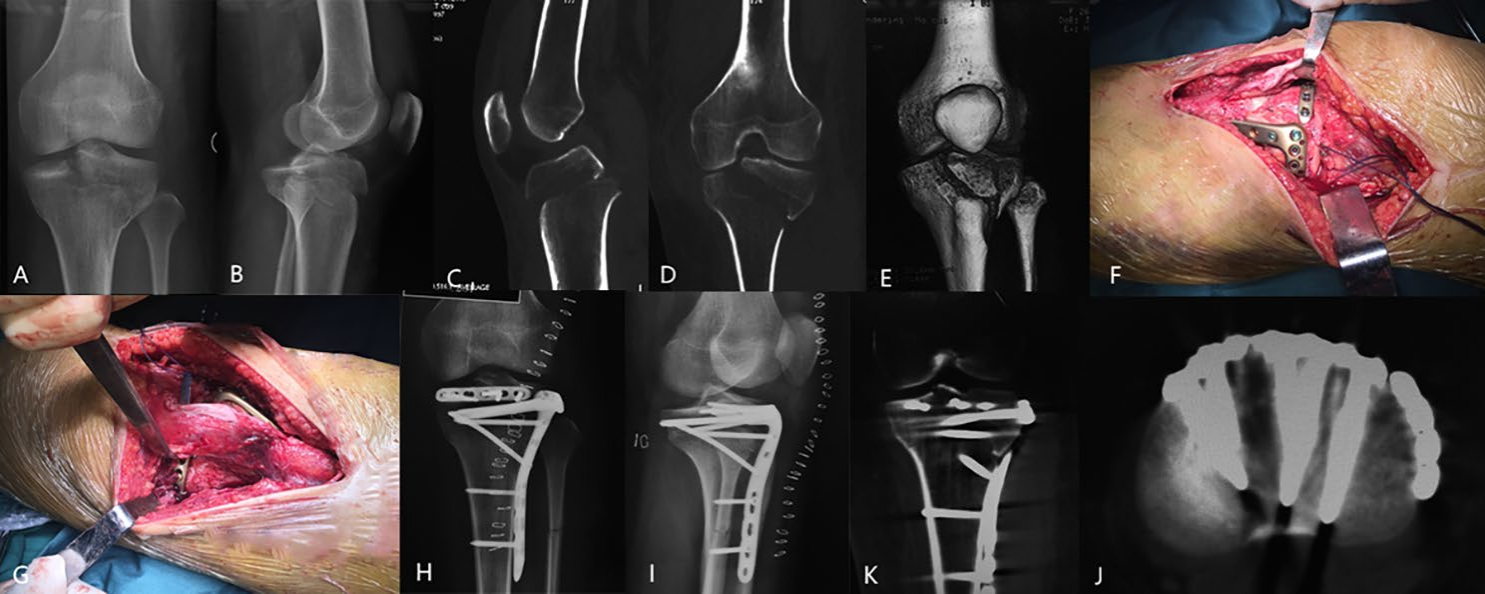
A 23-year-old female patient presented with lateral HTPF **(A-E)**. Subsequently, open reduction and fixation were carried out using a combination of the rim plate and anterolateral locking plate **(F-I)**. The screws of the rim plate were strategically placed by lifting both sides of the patella **(F, G)**. Postoperative CT scans demonstrated the successful provision of sufficient support to the anterior plateau **(J, K)**

For the fixation of the anteriorly reduced plateau, we initially employed anteromedial and/or anterolateral locking plates (Synthes GMBH, Oberdorf, Switzerland). Furthermore, to reinforce the “bare area” located behind and in close proximity to the patella tendon, a 2.7 mm plate (Synthes GMBH, Oberdorf, Switzerland) was pre-contoured to adapt to the curve of the anterior plateau. This plate was then inserted behind the patella tendon, above the locking plate. Subsequently, screws of suitable length were inserted on one or each side of the patella tendon, parallel to the articular surface. Additionally, at least one long screw was positioned in the posterior fragment or the uncomminuted medial/lateral fragment to prevent any secondary loss of reduction (Figs. 2 and 3).

### Postoperative management

Postoperative radiographs and CT scans were obtained to assess the surgical outcome. Early after surgery, passive and active rehabilitation, including a full range of motion (ROM), was initiated. Typically, non-weight bearing was maintained for 4 to 6 weeks, followed by partial-weight bearing. Full-weight bearing was permitted only after radiographs confirmed bone healing. The medial tibial plateau angle (mTPA) and PSA were measured on standard radiographs at both immediate post-operation and the last follow-up by two authors (Zhijian Sun and Changrun Li). Major complications were recorded, and clinical outcomes were evaluated using the hospital for special surgery (HSS) knee score at the last follow-up.

## Results

A total of 14 patients with HTPF underwent surgical treatment with rim plates. However, one patient was lost to follow-up, leaving 13 patients for the final analysis. The average follow-up period was 4.3 ± 1.5 (range: 1.9–6.5) years. The demographic data are presented in Table 1, comprising 6 males and 7 females with an average age of 45.1 ± 16.6 (range: 23–76) years. Bicondylar was involved in 9 patients, medial condylar in 3 patients, and lateral condylar in 1 patient. Schatzker’s classification and OTA classification was illustrated in Table 1. All patients received open reduction and rim plate combined with proximal tibial locking plate fixation. The average operation time was 101.2 ± 22.2 (range: 60–150) minutes, and the average intraoperative blood loss was 105.4 ± 69.8 (range: 20–250) mL.

**Table 1:** The demographic, surgical, and clinical data of patients

Number	Gender	Age (year)	Fracture type	Schatzker classification	OTA classification	Surgical time (minutes)	Intraoperative blood loss	Range of motion	HSS knee score
1	male	39	bicondylar	V	41-C3	120	100	0-130	98
2	female	23	Lateral condylar	I	41-B1	105	100	0-130	98
3	male	58	bicondylar	V	41-C3	120	250	0-120	97
4	female	34	bicondylar	V	41-C3	150	100	0-130	98
5	male	42	Medial condylar	IV	41-B3	90	20	0-125	92
6	female	55	Medial condylar	IV	41-B3	60	50	0-130	98
7	male	76	bicondylar	V	41-C3	100	200	0-130	98
8	female	27	Lateral condylar	II	41-B3	90	50	0-130	98
9	female	32	bicondylar	V	41-C3	90	200	5-120	96
10	female	25	bicondylar	V	41-C3	90	50	0-130	91
11	male	61	bicondylar	VI	41-C3	90	100	0-100	90
12	Female	55	bicondylar	V	41-C3	120	100	0-130	98
13	male	59	Medial condylar	IV	41-B3	90	50	0-130	98

OTA, Orthopaedic Trauma Association; HSS, hospital for special surgery

All patients achieved satisfactory reduction, which was radiographically maintained throughout the follow-up period. The medial tibial plateau angle (mTPA) measured 86.7°±1.9° (range: 83.9°-90.7°) immediately after surgery and 86.1°±2.7° (range: 82.7°-90.9°) at the last follow-up. Likewise, the medial PSA)and lateral PSA were 11.8°±4.1° (range: 4.3°-19.3°) and 12.0°±4.1° (range: 3.0°-16°) immediately after surgery, respectively, and changed to 6.8°±4.8° (range: -2.1°-14.6°) and 8.5°±6.4° (range: -2.9°-19.4°) at the last follow-up. Although there was a mild secondary loss of reduction, it did not lead to malalignment or articular depression. At the last follow-up, a satisfying clinical outcome was observed with a mean HSS knee score of 96.2 ± 2.0 (range: 90–98). No patients complained infrapatellar pain and all the patients could kneel painlessly.

One patient (patient no. 11) presented with preoperative nervus peroneus communis injury. A nerve graft was performed by a microsurgeon one year later, but ankle dorsiflexion did not significantly improve at the last follow-up. Nevertheless, the patient was able to walk without assistance. Throughout this patient cohort, no wound complications or implant breakages were observed.

## Discussion

HTPF represents a severe form of tibial plateau fracture, often linked to more frequent combined injuries and inferior functional recovery when compared to non-hyperextension types [4–8]. In clinical practice, the “bare area” is observed when only proximal tibial locking plates are used, which has been associated with a higher likelihood of secondary loss of reduction (Fig. 1) [9]. In this study, we present a novel approach utilizing the rim plate to support the anterior “bare area” and achieved satisfactory results in a case series.

The use of rim plates in the treatment of tibial plateau fractures is not uncommon, owing to their adequate buttressing strength and relatively small space occupation. Pires et al. [11] reported on 9 patients with posterolateral fractures treated through a transfibular approach, where rim plates were utilized to buttress the articular surface with a 2.7 mm horizontal rafting plate. Cho et al. [12, 13] described rim plating of posterolateral fractures using a modified anterolateral approach to achieve sufficient purchase. Zhu et al. [14] reported a new modified Frosch approach, using the “Barrel hoop plate” as a variant type of rim plate to fix the posterolateral fracture. Kumar and colleagues [15] presented a case of a patient with posteromedial tibial condyle fracture treated with a rim plate. Giordano et al. [16] introduced a new surgical technique for treating posterior bicondylar shear fractures using the “Hoop” plate. Furthermore, rim plate fixation could also be carried out minimally invasively with arthroscopic assistance [17].

In cases of HTPF, the extent of articular decompression typically covers more than half of the entire plateau. Additionally, anterior marginal fragment or decompression could be also involved, which includes the area behind the patella tendon. The standard proximal tibial plate alone may not offer adequate support for this entire region. Hence, the combined use of a pre-contoured rim plate serves as a reasonable option to provide extra subchondral support. In our series, we observed mild secondary reduction loss, which was nevertheless within an acceptable range. Further studies are required to verify the biomechanical advantages of the approach presented here.

One potential concern associated with this novel surgical strategy is the possibility of plate irritation on the patella tendon. However, the infrapatellar fat pad may serve as a buffering agent, and notably, no patients reported experiencing pain in the anterior region of the knee. Another aspect of concern was the biomechanical support strength of the rim plate, particularly considering the presence of comminuted fragments at the anterior plateau. To solve this problem, at least 1 long screw on the rim plate was inserted targeting the relatively intact posterior, medial, or lateral fragment. This additional reinforcement aimed to enhance the overall stability and support of the fixation.

The present study had several limitations. Firstly, a control group was not included in this case series, making it necessary for a comparative study to be performed in the future to verify its advantage. Additionally, a preoperative MRI was not routinely performed, so the accompanying meniscus and ligament injuries were not evaluated. Moreover, regular CT scans were not taken during the follow-up period, potentially resulting in the oversight of subtle secondary loss of reduction on radiographs.

## Conclusion

A novel fixation method for treating HTPF, the rim plate in combination with proximal tibial locking plates, provided adequate support strength to the anterior “bare area”. The presented case series demonstrated satisfactory clinical outcomes, with no major complications and minimal observable secondary reduction loss over a mean follow-up period of 4.3 years. However, rigorous designed comparative studies were still needed to further evaluate its effect.

## Data Availability

The data and materials can be obtained by contacting the corresponding author via email.

## Abbreviations

HTPF: Hyperextension tibial plateau fractures
CT: Computed tomography
MRI: Magnetic resonance imaging
PSA: Posterior slope angle
ROM: Range of motion
mTPA: medial tibial plateau angle
HSS: Hospital for special surgery
